# The utilization of Innovative, Eco-friendly recycled walls in the development of border regions’ educational buildings in Egypt

**DOI:** 10.1038/s41598-025-24113-5

**Published:** 2025-11-12

**Authors:** Mahmoud Yasser Ouf, Mohamed Gamal El-Kaissouny, Islam M. Nabil, Mohamed M. Mahdy

**Affiliations:** 1 Architectural Engineering Department, Helwan University, Helwan, Egypt; 2 Vice President of the General Authority for Educational Buildings, Cairo, Egypt; 3 Physics Department, Faculty of Science, Fayoum University, Fayoum, Egypt; 4 Architectural Engineering Department, Military Technical College, Cairo, Egypt

**Keywords:** Sustainability, Plastic bricks, Thermal comfort, Recycling, Reassembly, Engineering, Civil engineering

## Abstract

The construction sector plays a critical role in shaping social, economic, and environmental outcomes. It consumes about 19% of global energy and nearly half of the electricity used for building construction and operation. As a result, buildings are responsible for roughly one-third of global CO₂ emissions across their entire life cycle—from construction to operation and eventual demolition-making the sector a major driver of climate change and rising temperatures. For this reason, we argue that effective building design cannot be reduced to aesthetics or abstract concepts. It requires a holistic approach that combines thoughtful material selection, strategies to lower energy demand, and sustainable practices such as recycling. Passive design measures—such as optimizing building orientation, natural ventilation, and insulation-are particularly important because they reduce reliance on mechanical systems. In Egypt, however, external walls are still commonly built using half-brick red walls (12 cm thick), a low-cost solution with very poor thermal performance. While this practice keeps initial project costs down, it often undermines thermal comfort for residents and increases long-term energy use. In this study, we examine an alternative wall system: lightweight plastic bricks made from recycled material. These detachable blocks are compared with traditional red bricks to evaluate both the initial and operating costs. Our aim is to test whether such systems can improve indoor thermal comfort while remaining cost-effective under current and future climate conditions.

**Supplementary Information:**

The online version contains supplementary material available at 10.1038/s41598-025-24113-5.

## Introduction

 Energy conservation is a cornerstone of sustainable development worldwide, and it is especially critical in Egypt, where resource pressures and environmental challenges continue to grow. The building sector alone consumes nearly 40% of national energy, mainly due to heavy reliance on heating, ventilation, and air-conditioning (HVAC) systems in response to high temperatures^1^. This dependence not only drives energy demand but also intensifies urban heat islands and places additional stress on the energy grid ^2^.

The country’s vulnerability became clear during the electricity shortages of 2012 and 2013, when severe heat waves disrupted the national system ^3^. In response, the government launched several initiatives, including the 2008 “Egyptian Energy Code” ^4^, to promote energy efficiency in construction and guide designers and policymakers toward sustainable practices.

A key focus of these efforts is the building envelope—the interface between a building’s interior and the outdoor environment ^5^. The envelope regulates heat transfer through walls, roofs, and windows, making it central to reducing energy demand and improving indoor comfort. When combined with passive solar design strategies, innovative materials can significantly improve thermal performance while reducing reliance on mechanical systems ^6^.

In Egypt’s border regions, these needs are even more urgent. Buildings must be cost-effective, easy to assemble and maintain, and energy-efficient, given the unique socio-economic and environmental conditions. This creates demand for materials that are lightweight, durable, recyclable, and adaptable ^7^.

In this study, we explore whether recycled plastic bricks can provide a viable alternative to traditional red bricks in educational buildings. Using Design-Builder software, we simulate the thermal performance of both materials under realistic conditions based on Egypt’s Typical Meteorological Year (TMY) data ^8^. The objectives are threefold: to assess thermal and energy performance, to evaluate cost-effectiveness and indoor environmental quality (IEQ), and to test the feasibility of innovative, recyclable materials for rapid construction in border areas. We also validate the simulation results through experimental comparisons to ensure practical applicability ^9^. Beyond Egypt, the findings have broader relevance. The study demonstrates how sustainable, flexible construction materials can address challenges in regions facing energy scarcity, climate change, or the need for temporary and mobile buildings. By integrating advanced materials with innovative design strategies, we aim to provide actionable insights for designers, policymakers, and stakeholders, while emphasizing the importance of balancing environmental, economic, and social considerations ^10,11^.

**Fig. 1:**
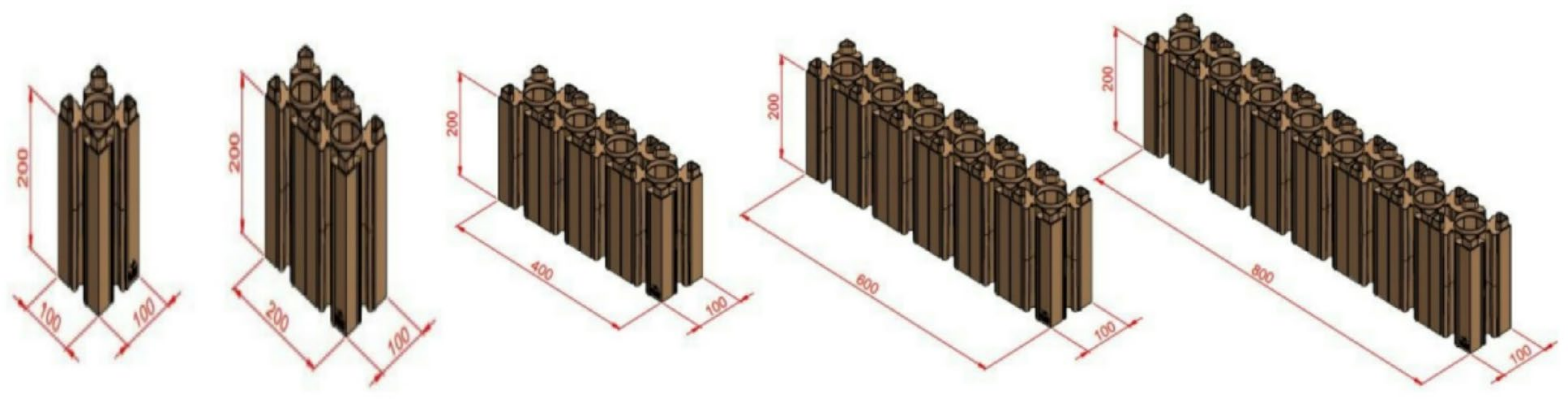
The dimensions of the innovative plastic bricks *Source Design-Builder (Version 7) *^38^.

In this study, we evaluate the effectiveness of alternative wall materials in reducing thermal loads and long-term operational energy costs. To achieve this, we conducted a comparative analysis between conventional red bricks and recycled plastic bricks, testing their performance under projected climatic conditions extending to 2080. The results highlight several advantages of recycled plastic bricks. They improve energy efficiency, reduce initial construction costs, and allow for faster installation. Their modular design also supports quick assembly and disassembly, which makes them especially suitable for sustainable and flexible construction. These qualities are particularly valuable in resource-constrained settings, where efficiency, adaptability, and affordability are essential. as illustrated in Fig. 1.

## The general structure of the article

Given Egypt’s hot and arid climate, buildings have traditionally relied on thick masonry walls to delay heat transfer from the harsh exterior environment ^32^. Nonetheless, the half red brick wall—12 cm thick with low thermal insulation—remains pervasive due to its low cost and availability ^37^. While economical upfront, this approach contributes to excessive energy consumption, poor indoor comfort, and high operational costs over the long term.

### Eco-friendly alternative wall materials

Recent research is exploring sustainable walling alternatives that balance cost, durability, and thermal performance. For example, plastic-waste–reinforced cement bricks show improved thermal resistance and strength when high-density polyethylene (HDPE) or polyethylene terephthalate (PET) partially replace aggregates, reducing thermal conductivity and enhancing building insulation ^29^. Similarly, lightweight concrete blocks incorporating recycled brick and polystyrene aggregates have demonstrated superior energy savings and occupant comfort in renovation applications ^30^. Another promising area is geopolymer insulation bricks synthesized from industrial waste, which deliver excellent eco-benefits while maintaining insulation performance ^33^.

### Modular & recyclable systems for remote educational buildings

For remote or border-region schools, flexibility and rapid deployment are critical. Studies in rural India and sub-Saharan Africa emphasise modular panels made from locally available recycled materials, enabling fast assembly and dismantling—similar in concept to the “plastic brick” system ^36^. While not directly in the Egyptian context, these cases underscore the practicality of recyclable modular systems for educational infrastructure in challenging regions.

### Comparative studies of alternative wall systems

Comparative analyses provide valuable insights: rammed earth and compressed earth block (CEB) walls offer high thermal mass and low embodied energy, reducing heating and cooling loads significantly—by 20–52% compared to conventional assemblies ^34^. Systematic reviews further confirm that stabilised CEBs and rammed earth constructions achieve mechanical strengths up to 24 MPa while exhibiting a markedly lower environmental impact ^31^. Life-cycle comparisons of natural wall assemblies—such as cob, light straw clay, and rammed earth—versus clay or concrete block walls also reveal substantial sustainability gains when using bio-based insulation ^35^.

### Positioning this study within the literature

Despite these advances, few studies address all of the following: (i) recycled plastic modular walls, (ii) rapid deployment and dismantling, and (iii) integration into educational buildings located in hot desert or border environments. Our research fills this gap by introducing a lightweight, modular plastic brick system—reinforced with polypropylene connectors and recycled organic fibers—adapted for Egyptian border-region classrooms. Unlike previous studies focusing on thermal performance or recycling alone, we present a comprehensive framework combining simulation-based thermal analysis, structural efficiency, economic evaluation, and field applicability.

## Materials and methods

The central aim of this research is to evaluate the application of plastic-based walls as an innovative solution for building envelopes, with particular attention to their performance under projected climate change scenarios. To address this objective in a systematic manner, the methodology has been restructured into four main subsections: material preparation, experimental design, testing procedures, and data analysis ^8,15,18^,].

### Context and framework

The methodology was structured to evaluate recycled plastic bricks as a sustainable alternative to conventional red bricks in educational buildings located in border regions. It encompasses four core components: material development, experimental simulations, prototype testing, and environmental as well as economic analysis. Each stage was carefully aligned with the overarching research objective—assessing the energy performance, cost-efficiency, and environmental benefits of the proposed system.

### Material preparation

**Fig. 2:**
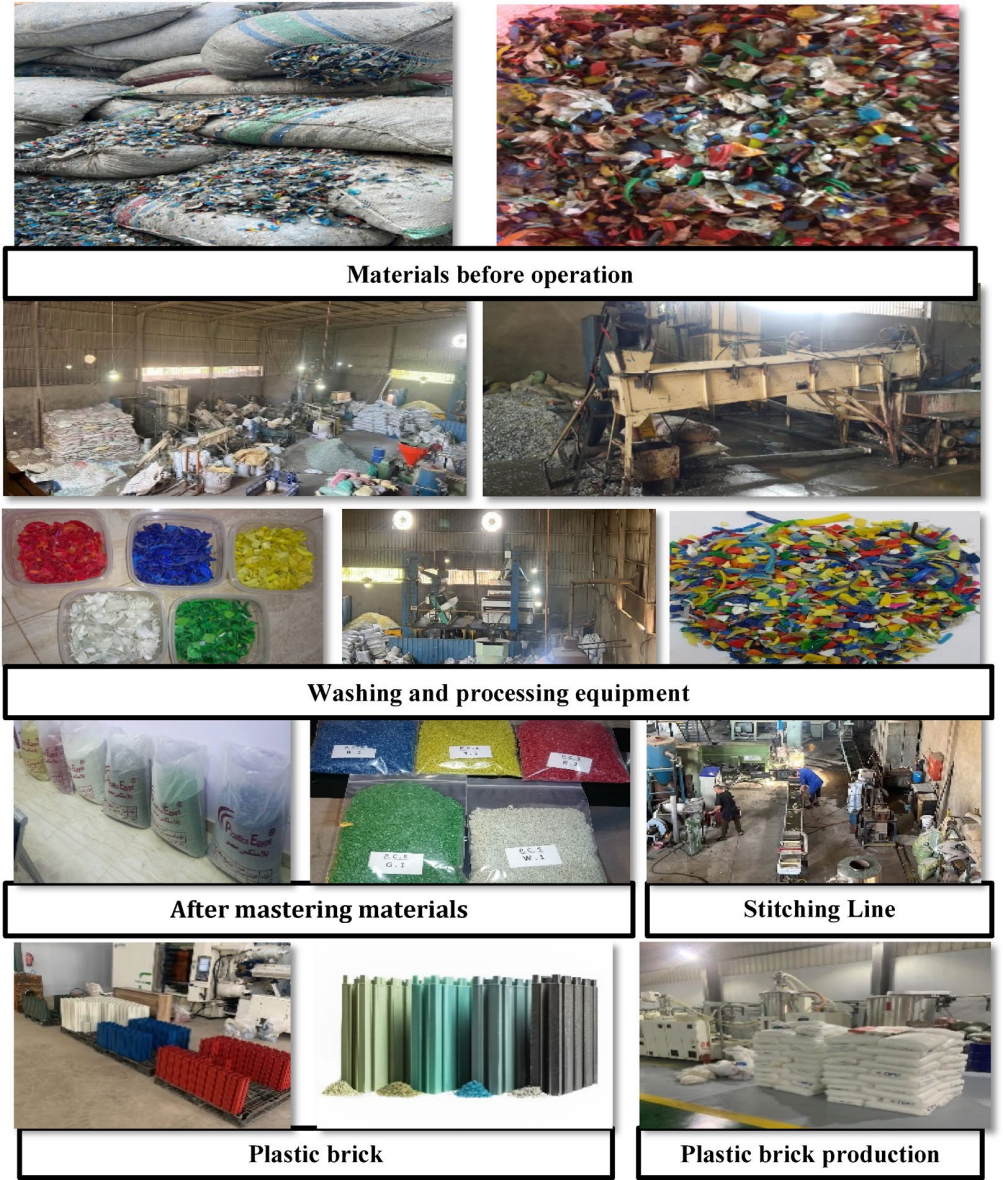
Preparation of plastic bricks using the raw input materials.

Recycled plastic bricks were produced using three primary waste streams: polyethylene terephthalate (PET) from bottle bodies, polypropylene (PP) from labels, and polyethylene (PE) from caps. The manufacturing process followed a standardized five-step sequence consisting of breaking, washing, material separation, granulation, and compression molding ^14,22^. The resulting HDPE/LDPE composite units were further reinforced with polypropylene connectors and recycled organic fibers to enhance structural stability and durability ^7,11^. Each unit measured 30 × 15 × 15 cm, dimensions comparable to conventional masonry blocks (Fig. 2). The physical properties of the developed bricks included an average density of 920 kg/m³ and a thickness of 0.15 m. Thermal conductivity values were calibrated and validated against prior experimental studies ^14^.

### Material selection criteria

The selection of materials was guided by explicit criteria, namely cost efficiency ^9^, availability in border regions ^15^, thermal performance  ^5,17^, environmental impact ^16,22^, and durability/reusability ^7,11^.

These parameters were particularly relevant to the challenges of educational facilities in border areas, where projects often face strict budget constraints, remote site conditions, climatic stresses, and ambitious sustainability goals ^6,7,9^. Cost efficiency was prioritized to minimize both initial construction expenses and long-term operational costs, a critical factor in publicly funded border-region schools ^9^.

In parallel, local or regional availability of materials was emphasized to reduce transportation costs and logistical barriers, in line with Egyptian guidelines for construction in remote areas ^15^.

 Thermal performance was another essential criterion, as adequate insulation is required to ensure indoor comfort under the country’s diverse climatic zones, in compliance with ASHRAE standards and the givoni comfort chart ^5,17^.

Furthermore, the environmental impact of construction materials was assessed with reference to the ICE database and Egypt’s Energy Efficiency Code, highlighting the advantages of recycled plastics in reducing embodied energy and carbon emissions compared to conventional red bricks ^16,22^. Finally, durability and reusability were considered fundamental in light of desert climatic conditions and the need for temporary or relocatable facilities, where materials must withstand harsh stresses while allowing for future disassembly and reuse ^7,11^.

Collectively, these criteria align with previous studies on sustainable material selection for educational buildings ^6,7,9^ and supported the choice of recycled plastic bricks reinforced with polypropylene connectors and organic fibers.

### Experimental design

**Fig. 3:**
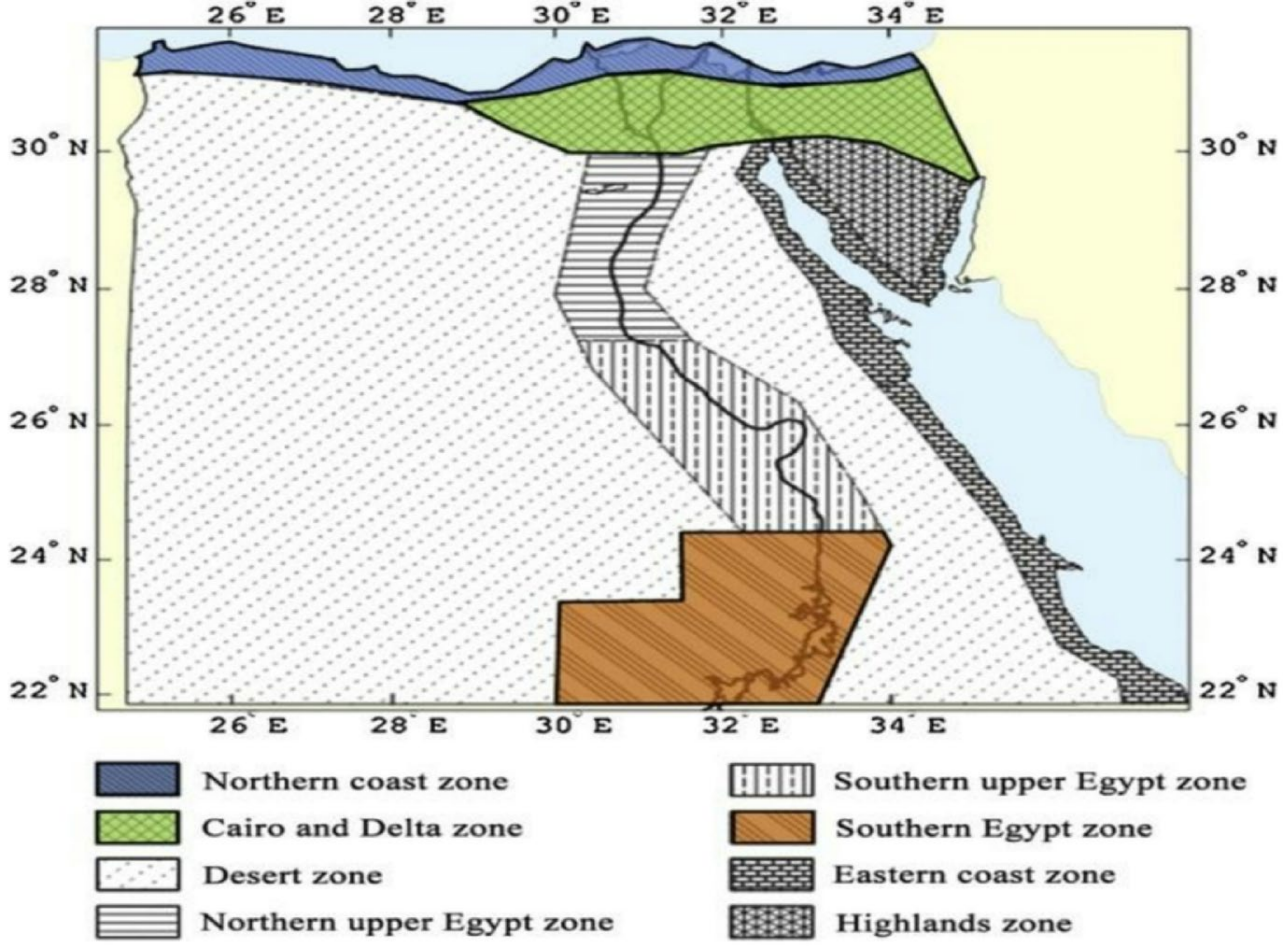
Climate zones in Egypt as defined by EREC Source: Mahdy et al., 2017.

A standardized classroom model provided by the General Authority for Educational Buildings was adopted as the case study ^3^, as shown in Fig. (3).

Two design prototypes were modeled for comparative analysis:

the first constructed with conventional red bricks (12 cm thickness, 1800 kg/m³ density) and the second employing modular recycled plastic bricks.

The simulations were conducted across three representative climatic zones in Egypt—Alexandria (moderate), Cairo (hot semi-arid), and Aswan (hot arid)—in accordance with the EREC classification ^15^. Thermal performance was analyzed using Design-Builder software, which incorporated typical meteorological year (TMY) weather data specific to each location ^20^. The operational profile reflected real classroom conditions, with 40 students occupying the space for seven hours daily (08:00–15:00), five days per week. Internal heat gains were set at 70 W/person, 15 W/m² for lighting, and 10 W/m² for equipment loads ^19^.

Indoor air temperature was employed as the primary thermal comfort indicator and was further validated using the Givoni bioclimatic chart ^17^.

This study investigates a standardized classroom model developed by the General Authority for Educational Buildings—the government agency responsible for constructing public schools in Egypt. The case study was situated in Aswan, one of Egypt’s border regions, selected due to its educational importance and relevance to the project’s objectives. The chosen model represents a typical classroom frequently implemented in public schools to address students’ learning needs.

**Fig. 4:**
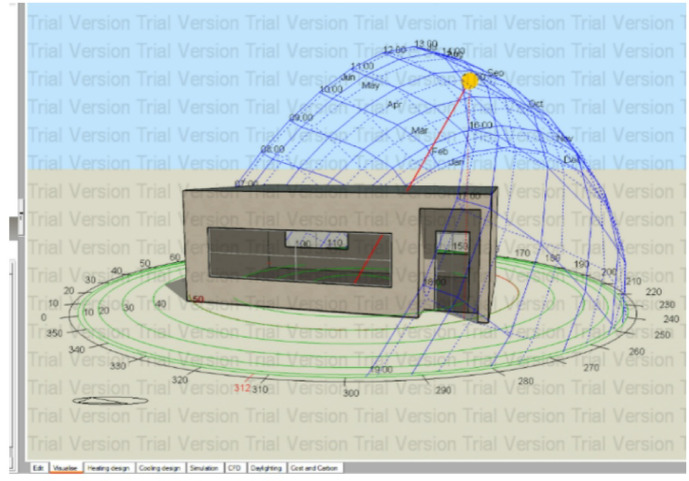
Screenshot from Design-Builder (Version 7) interface showing energy performance simulation.

Two building prototypes were simulated, each consisting of a single ground floor with identical window-to-wall ratios, orientations, and envelope configurations, as illustrated in Fig. (4). The comparative analysis focused on two wall systems:

(1) conventional red brick walls.

(2) demountable recycled plastic bricks—lightweight, reusable units reinforced with polypropylene connectors and recycled organic fibers ^3^.

These prototypes served as the basis for both structural and environmental performance assessment.

### Testing procedures

- Structural analysis: ETABS 2016 and SAFE 2016 were applied to columns, slabs, and beams, quantifying savings from reduced dead load ^8,18^.
- Prototype construction: A classroom was built using recycled plastic bricks, requiring ~ 4 days compared to ~ 80 days for red brick masonry. Reassembly confirmed modularity ^6,7^.
- Laboratory tests: Conducted at the National Research Center: compressive strength, fire resistance (> 60 min at 140 °C, up to 1260 °C furnace) ^24^, and acoustic insulation (~ 30 dB) ^23^.
- Thermal performance: Indoor air temperature reduction of ~ 1.5 °C for plastic bricks vs. 0.3 °C for red bricks, corresponding to ~ 15–18% cooling energy savings ^3,21^.

*Design-Builder* software was used for thermal and environmental simulations, while ETABS and SAFE were used for structural analysis.

A prototype classroom was constructed with recycled plastic bricks, and laboratory tests were carried out at the National Research Center.

These procedures were chosen to combine predictive simulations with empirical validation, ensuring robust evaluation of the recycled plastic system ^6–8,11,15^.

A comparative case study was conducted to assess the performance of modular plastic brick walls relative to conventional red brick walls. The significantly lower structural mass of recycled plastic bricks contributes to lighter foundations, smaller column dimensions, and thinner slab requirements. This reduction translates into decreased consumption of construction materials and lower structural costs ^8^. Specifically, the analysis focused on savings in both ordinary and reinforced concrete, which result from the reduced dead load—the permanent weight of structural components such as walls, floors, and roofs—offered by detachable and re-mountable lightweight plastic brick systems.

**Fig. 5:**
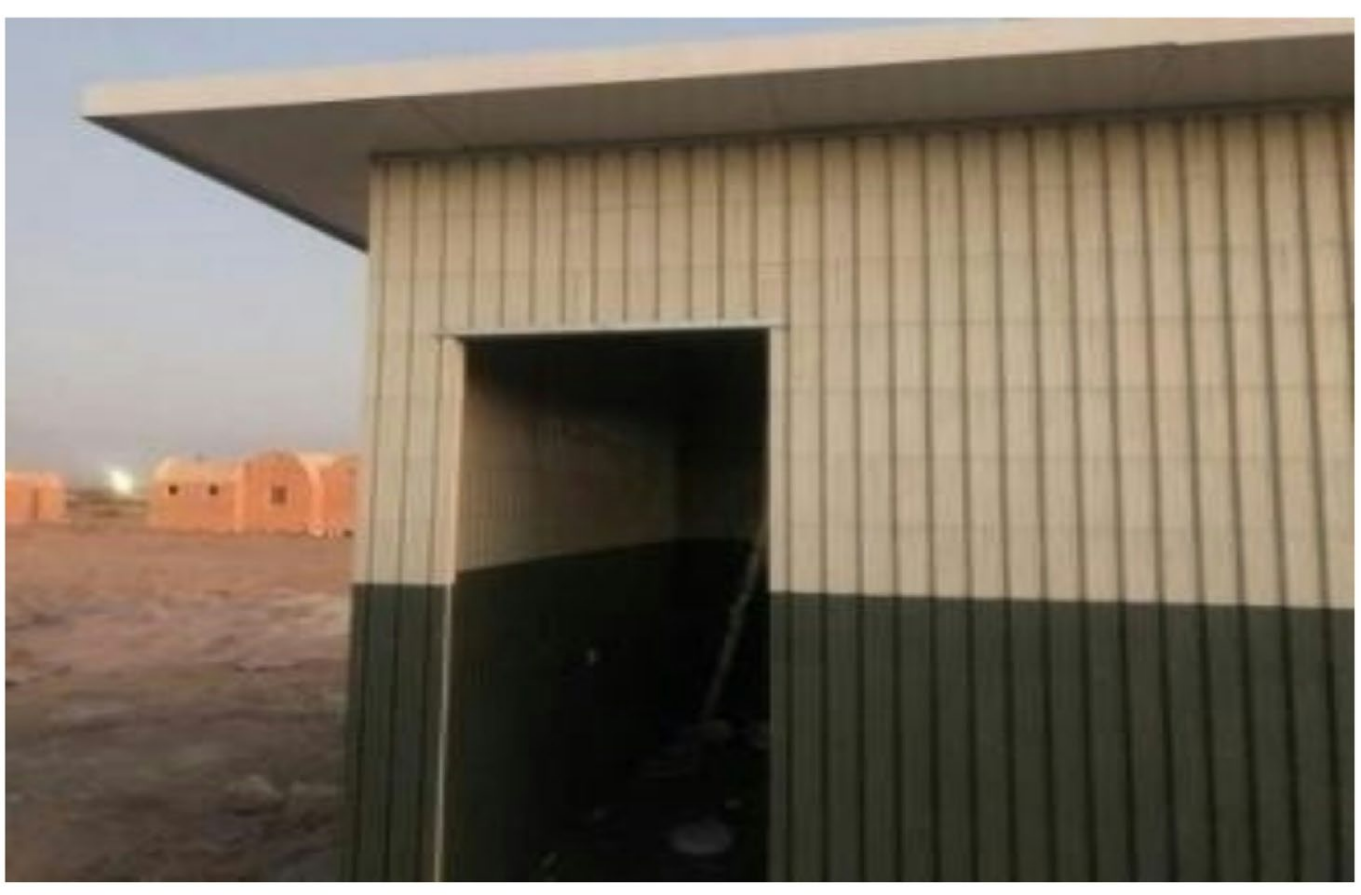
Actual construction progress at the case study site.

**Fig. 6:**
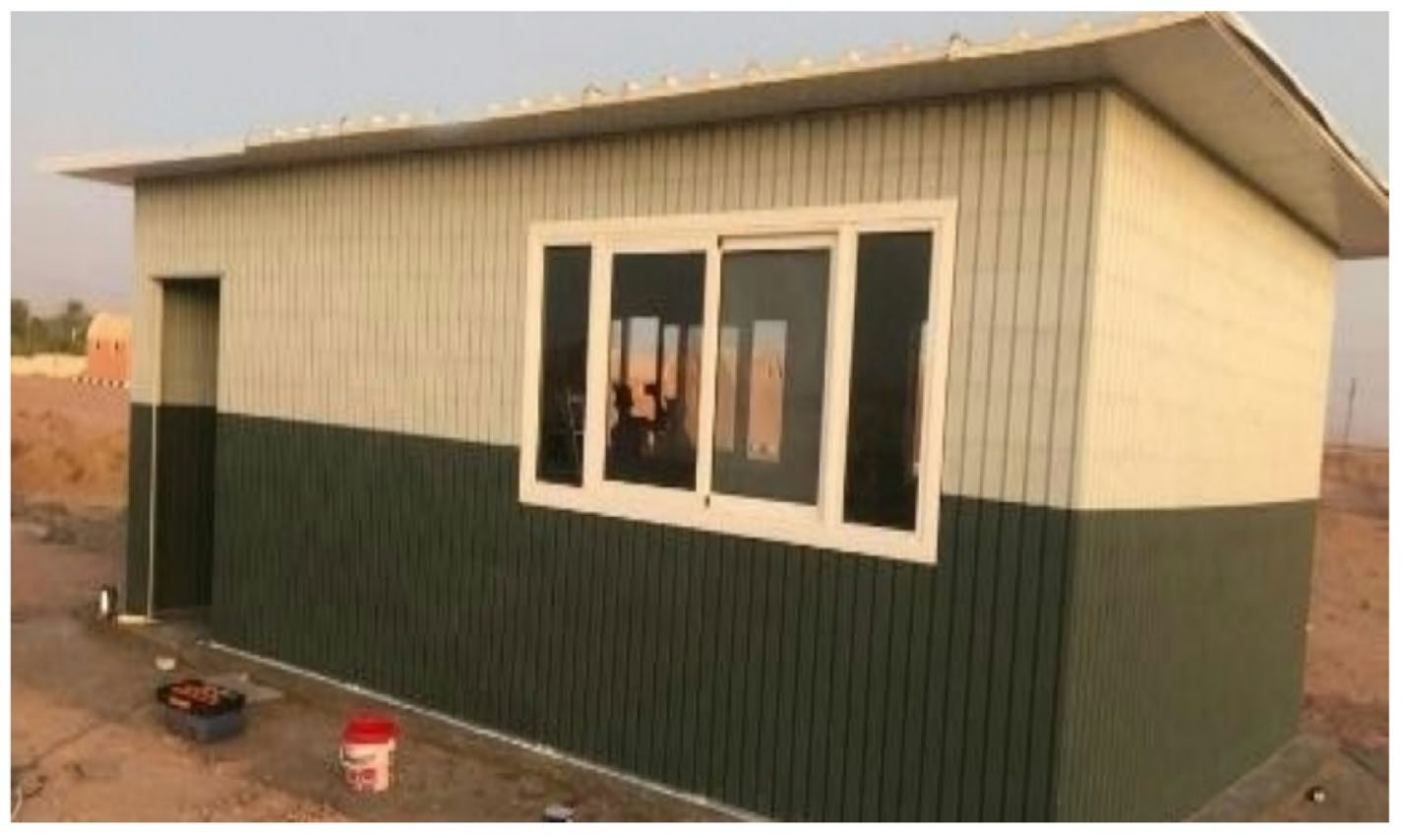
Actual construction progress at the case study site.

A pilot classroom was constructed using the proposed recycled plastic bricks to evaluate their real-life feasibility. Field testing validated the system’s ease of installation, rapid construction process, and structural durability under actual site conditions ^6^. The assessment also considered the potential for transportation, disassembly, and reinstallation, demonstrating the modular adaptability of the system, as illustrated in Figs. (5) and (6).

In addition to the laboratory-based physical and mechanical tests, a pilot building was constructed using the proposed recycled plastic bricks to demonstrate the feasibility of real-life applications. This prototype was employed to evaluate the assembly process, structural integrity, and practical installation performance under actual site conditions. The field pilot testing confirmed the system’s durability, ease of installation, and potential for rapid construction, thereby complementing the simulation outcomes with empirical evidence. These findings were further supported by experimental validation conducted at the National Research Center, as illustrated in Figs. (A1–A3).

**Fig. 7:**
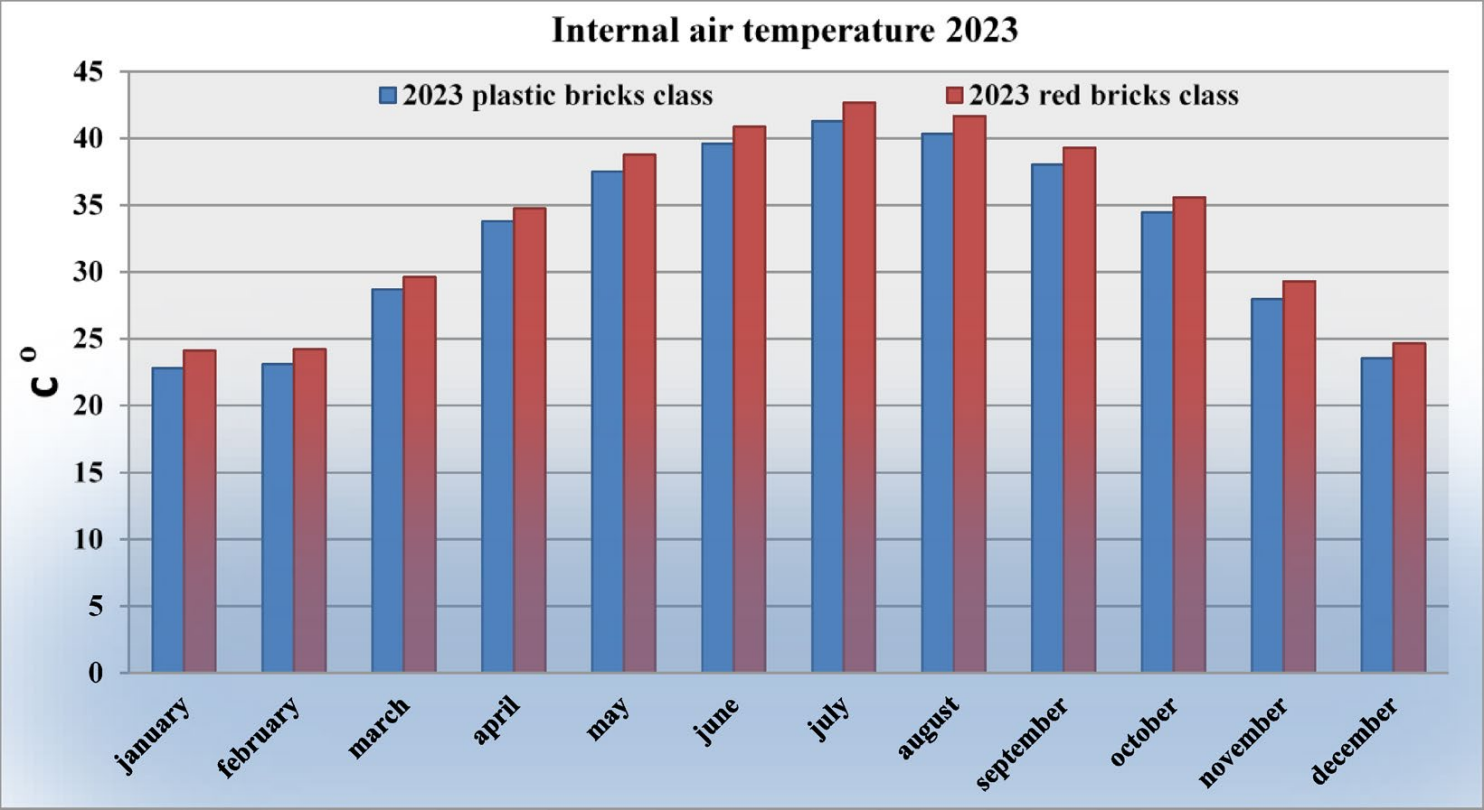
Comparison of indoor temperatures for walls built with traditional red brick and with removable plastic bricks reinforced with polypropylene and recycled organic fibers, measured during the same period in 2023.

Experiments and simulation-based analyses were conducted to evaluate the temperature variations associated with different wall materials. The results indicated that classrooms constructed with recycled plastic bricks achieved an average indoor temperature reduction of approximately 1.5 °C, compared to only 0.3 °C for conventional red brick walls. This reduction translates into lower electrical demand for mechanical ventilation and space cooling, thereby enhancing energy efficiency. The simulations were carried out using the Design-Builder software, which had been previously calibrated and validated to ensure accuracy. Indoor air temperature profiles were compared for both wall types over the same operational period, as illustrated in Fig. 7 ^21^.

Fig. 7 illustrates the comparison of indoor air temperatures between classrooms constructed with conventional red brick walls and those built with demountable recycled plastic bricks reinforced with polypropylene connectors and recycled organic fibers, during the year 2023. Complementarily, Fig. 9 presents a bar chart summarizing the monthly temperature variations across the twelve months. The results reveal a consistent trend:

classrooms with recycled plastic brick walls maintained lower indoor temperatures than their red brick counterparts, with the temperature gap becoming more pronounced during the warmer months from June to September.

The analysis indicates that although recycled plastic bricks are primarily composed of thermoplastic materials, their engineered formulation—integrated with polypropylene reinforcement and recycled organic fibers—significantly improves thermal stability under fire exposure.

The experimental results confirmed that the units maintained structural integrity for more than 60 min under direct flame, a performance particularly critical for educational buildings in Egypt’s border regions, where rapid evacuation and occupant safety are essential. These results are summarized in Table 1.

The performance of recycled plastic brick walls aligns with international safety guidelines for non-structural partition systems, thereby supporting their application in classrooms and temporary facilities ^11^.

**Fig. 8:**
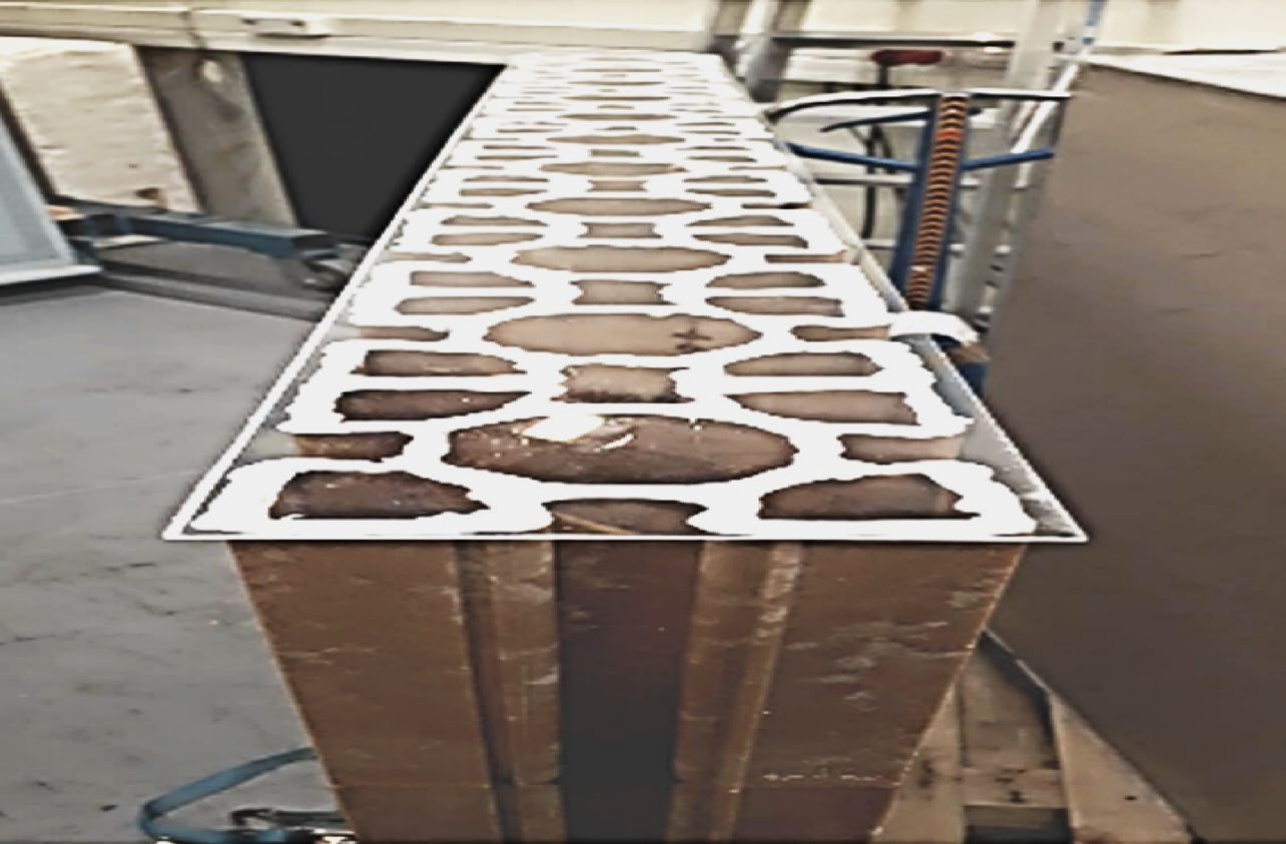
Plastic brick samples after fire resistance test.

**Fig. 9:**
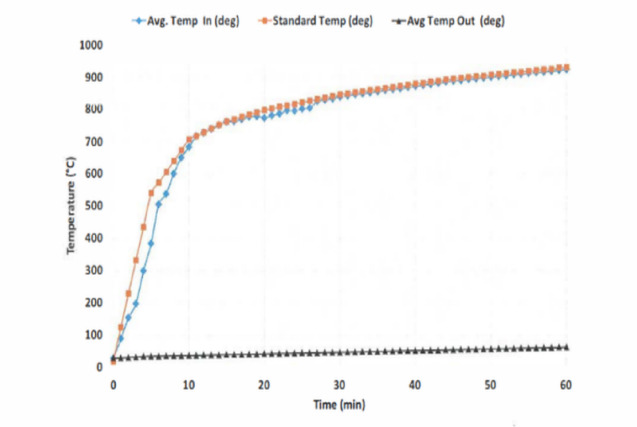
Variation of furnace temperature with heating time during fire resistance testing.

Fire resistance tests were conducted at the Housing and Building National Research Center, with the experimental setup illustrated in Figs. 8 and 9.

Table 1 presents the variation of furnace temperature (°C) with heating time (minutes). The results demonstrate a gradual temperature increase over intervals up to 480 min, ultimately reaching a maximum of 1260 °C.

**Table 1:** Variation of furnace temperature with heating time during fire resistance testing.

Time (min)	5	10	30	60	120	240	480
Furnace temperature (c)	538	704	843	927	1010	1093	1260

The figure presents the results of fire resistance tests on the recycled plastic brick system, carried out at the Housing and Building National Research Centre.

The tests evaluated the material’s behaviour under direct flame exposure across different heating durations and corresponding furnace temperatures. The chart illustrates that the recycled plastic brick system retains its structural integrity up to approximately 140 °C, achieving more than one hour of continuous fire resistance before the onset of melting. These results were obtained under standard testing conditions, where furnace temperatures progressively increased from 538 °C at 5 min to 1260 °C at 480 min. Although recycled plastic bricks are primarily composed of thermoplastic materials, their engineered formulation—reinforced with polypropylene and recycled organic fibers—significantly enhances thermal stability under fire exposure.

Maintaining integrity for over 60 min is particularly critical for educational buildings in Egypt’s border regions, where occupant safety and rapid evacuation are of paramount importance.

The performance complies with international safety guidelines for non-structural partition systems, supporting their application in classrooms and temporary educational facilities. Moreover, the demonstrated fire resistance complements the system’s other advantages—lightweight design, modularity, and energy efficiency—as highlighted in the previous figures. Collectively, these attributes confirm that the recycled plastic brick system can contribute to sustainable construction practices while meeting fundamental fire safety requirements. This integrated fire safety aspect strengthens the system’s potential for large-scale deployment in sustainable educational infrastructure, directly addressing stakeholder concerns about the flammability of plastic-based building materials.

### Data analysis

- Embodied energy & carbon: Calculated from ICE v3.0 database ^16^. Results expressed as MJ/m² and kgCO₂e/m², cradle-to-gate.
- Economic evaluation: Based on Q2–2023 Egyptian market data, adjusted for 2024 inflation (Central Bank of Egypt) ^25^.
- Sensitivity analysis: Thermal conductivity and density varied ± 10%; deviations in cooling loads remained within ± 5%, confirming robustness ^21,26^.

Embodied energy and carbon emissions were calculated using the ICE v3.0 database. Comparative analysis covered construction cost, implementation period, thermal insulation, and indoor comfort. Sensitivity analysis tested robustness by varying thermal conductivity and density by ± 10%. Results showed stable performance within ± 5% deviation, confirming reliability. This analytical approach was chosen to ensure transparency and reproducibility.

Embodied energy and carbon emissions for red brick and recycled plastic wall components were calculated using material unit values obtained from the ICE 3.0 database (University of Bath, 2019). The environmental impact per square meter of wall was estimated by multiplying the unit impact (MJ/kg and kgCO₂e/kg) by the corresponding material density and wall thickness. Only cradle-to-gate values were considered in this analysis ^3,16^, as illustrated in.Table 2.

**Table 2:** Embodied energy and carbon impact per 1 m² of external wall.

Wall Type	Material	Density (kg/m³)	Wall Thickness (m)	Mass per 1 m² (kg)	Embodied Energy (MJ/kg)	Total EE (MJ/m²)	Embodied Carbon (kgCO₂e/kg)	Total EC (kgCO₂e/m²)
Red Brick Wall	Fired Clay Brick	1,800	0.2	360	2.4	864	0.22	79.2
Recycled Plastic Wall	HDPE/LDPE Composite	920	0.15	138	8.1	1,118	1.75	241.5

- **Density and thickness** values are based on manufacturer data and design assumptions used in the thermal simulation model.
- **Embodied Energy (EE)** and **Embodied Carbon (EC)** values sourced from *ICE v3.0 (University of Bath*,* 2019)*.
- **Only cradle-to-gate** emissions considered (i.e., raw material extraction → manufacturing).

To complement the simulation-based environmental performance analysis, embodied energy and carbon emissions were calculated for the two wall construction systems: traditional red brick and recycled plastic wall blocks (Table 3).Material unit values were obtained from the ICE 3.0 database (University of Bath, 2019), a widely used source for life cycle environmental data. The environmental impact per square meter of wall surface was calculated using the following formula:

1 $$\begin{aligned} Impact \; (per m^{2}) & = Density \; (kg/m^{3}) \times Wall \; Thickness (m) \\ & \quad \times Embodied \; value (MJ/kg \; or \; kgCO_{2}e/kg) \end{aligned}$$

Only cradle-to-gate values were considered (i.e., emissions and energy from raw material extraction through to manufacturing), excluding transportation, use, and disposal stages ^16^ as illustrated in Table 3.

**Table 3:** Embodied energy and carbon impact per 1 m² of external wall.

Wall Type	Material	Density (kg/m³)	Wall Thickness (m)	Mass per 1 m² (kg)	EE (MJ/kg)	Total EE (MJ/m²)	EC (kgCO₂e/kg)	Total EC (kgCO₂e/m²)
Red Brick Wall	Fired Clay Brick	1800	0.20	360	2.4	864	0.22	79.2
Recycled Plastic Wall	HDPE/LDPE Composite	920	0.15	138	8.1	1,118	1.75	241.5

Density and thickness values are based on manufacturer data and design assumptions used in the thermal simulation model.

EE and EC values were sourced from ICE v3.0 (University of Bath, 2019).

Only cradle-to-gate emissions were considered in this assessment.

## Results and discussion

The simulation was carried out on the selected case study by comparing two external wall systems: (1) a conventional red brick wall, and (2) a modular, removable wall system constructed from recycled plastic bricks reinforced with polypropylene and recycled organic fibers. The comparative assessment focused on three key dimensions: thermal performance, environmental impact, and construction efficiency.

 A consolidated overview of these parameters is presented in Table 4^3^.

To strengthen the validity of the findings, statistical variability and uncertainty were systematically addressed. Standard deviations were calculated for indoor temperature and cooling energy demand across the three representative climate zones. Furthermore, a sensitivity analysis was performed by varying the thermal conductivity and density of wall materials by ± 10%, with the resulting changes in annual cooling loads documented in Table 4. This approach ensures robustness and reliability of the simulation outcomes^16^.

In addition to performance metrics, implementation indicators (such as ease of construction, modularity, and potential for reuse) are also summarized, providing a holistic evaluation of both wall systems, as shown in Table 4.

**Table 4:** The performance metrics.

Item	Traditional Brick Wall	Recycled Plastic Brick Wall
**Equipment Required**	Requires special handling equipment	Easy handling without special equipment
**Implementation Period**	~ 80 days	~ 4 days
**Plain Concrete Volume**	25 m³	9 m³
**Construction Cost**	EGP 450,000 (excluding border work)	EGP 180,000
**Construction Cost Savings**	–	Approx. 30%
**Electricity Bill Savings**	–	Approx. 60%
**Thermal Insulation Improvement**	Approximate reduction of 0.3 °C in indoor temperature	~ 1.5 °C indoor reduction
**HVAC System Requirement**	Required	Not required under typical climate conditions
**Transportation Logistics**	Requires special equipment	Simple and lightweight; easy transport
**Sustainability**	High fossil fuel consumption	Made from post-consumer recycled materials
**Reusability & Modularity**	Not reusable	Fully disassemblable and reconfigurable
**Environmental Impact**	Polluting and non-recyclable	Low-emission and recyclable
**Sound Insulation**	Poor acoustic performance	Improved sound insulation

All cost estimates were derived from actual local market prices in Egypt as of Q2 2023, based on quotations collected from five independent suppliers in Greater Cairo.

To ensure accuracy, prices were adjusted for inflation using the 2024 official rates published by the Central Bank of Egypt ^9^.

Mean values and standard deviations were calculated to capture market variability, thereby enhancing transparency and reproducibility of the economic analysis ^25^.

The analysis demonstrates that the proposed recycled plastic brick wall system achieves tangible savings in both construction costs and long-term operational energy consumption. Sensitivity testing further confirmed the robustness of these findings:

when thermal conductivity and density of wall materials were varied by ± 10%, total cooling energy demand fluctuated within a margin of only ± 5%, while the comparative advantage of the plastic brick wall remained consistent across all scenarios ^21^. On average, recycled plastic wall systems achieved a 15.7% reduction in cooling energy consumption relative to conventional red brick walls across the three studied climate zones. Performance remained within the ± 5% statistical significance threshold, reinforcing confidence in the material’s long-term energy-saving potential ^26^. To validate the simulation model, indoor temperature profiles were compared against empirical data reported by Fahmy et al. (2014), representing measured thermal conditions in public school buildings in Cairo. Model accuracy was evaluated using Mean Absolute Error (MAE) and Root Mean Square Error (RMSE), both of which indicated acceptable levels of deviation between simulated and measured data ^21^.

Collectively, this methodology—combining market-based cost data, inflation-adjusted prices, sensitivity analysis, and empirical validation—ensures that the reported findings are both statistically robust and practically meaningful.

## Conclusions

This study explores the potential of recycled plastic bricks as an alternative to traditional red bricks in educational buildings, with a focus on both economic and environmental performance.

From an economic perspective, the use of detachable plastic brick blocks—reinforced with polypropylene and recycled organic fibers—proved highly beneficial. By reducing the overall dead load of the structure, these bricks decrease the demand on foundations, columns, and slabs. As a result, the amount of reinforced concrete required is lowered, leading to an estimated saving of around 30% per cubic meter of concrete in wall construction.

The environmental and thermal performance assessments provide equally promising results. Simulations carried out with *Design-Builder* under three projected climate scenarios up to 2050 showed that buildings constructed with plastic bricks can lower indoor air temperatures by approximately 0.5–1.5 °C. Although this reduction alone does not achieve the ideal comfort range of 20–30 °C, it still helps ease cooling and heating demands. Consequently, electricity consumption and operating costs are reduced over the building’s lifecycle ^5^.

Another key advantage lies in construction efficiency. The lightweight nature and modular form of plastic bricks allow for building times to be shortened by up to 90% compared with conventional methods. Beyond speed, the use of recycled materials—such as discarded plastic bottles—supports waste reduction and contributes to a more circular and sustainable construction process.

To test the reliability of these outcomes, a sensitivity analysis was conducted on two critical parameters: thermal conductivity and material density. Both were varied by ± 10% under the Cairo climate scenario. The results showed only limited fluctuations in energy demand: within ± 4.7% for thermal conductivity and within ± 2.5% for density. This stability suggests that the relative performance advantage of plastic bricks remains consistent even when accounting for reasonable uncertainties in material properties ^9^.

Nonetheless, some limitations must be acknowledged.

While sensitivity ranges and descriptive statistics were applied, advanced uncertainty analyses, such as Monte Carlo simulations, were not undertaken. This restricts the full exploration of parameter variability and its potential influence on results ^27^.

Additionally, as the findings are primarily based on simulation, real-world outcomes may differ due to construction quality, user behavior, or site-specific conditions. To strengthen the reliability of the results, future research should incorporate field measurements and long-term monitoring ^26^. Finally, while the study demonstrates strong potential for sustainable and cost-effective educational infrastructure, large-scale implementation may encounter challenges. Issues such as supply chain readiness, compliance with local building codes, and cultural acceptance could affect adoption. Addressing these factors in future research will be critical to ensuring that plastic brick systems can be practically and widely applied ^28^.

## Supplementary Information

Below is the link to the electronic supplementary material.

Supplementary Material 1

## Data Availability

All data generated or analyzed during this study are included in this published article.
