# Supplementary material for: The utilization of Innovative, Eco-friendly recycled walls in the development of border regions’ educational buildings in Egypt
Source: Sci Rep. 2025 Nov 12;15:39549. doi: 10.1038/s41598-025-24113-5 (PMC12612244; doi:10.1038/s41598-025-24113-5)
Supplement: Supplementary file 1 — Supplementary Material 1 [file 41598_2025_24113_MOESM1_ESM.docx]

**Appendix:**


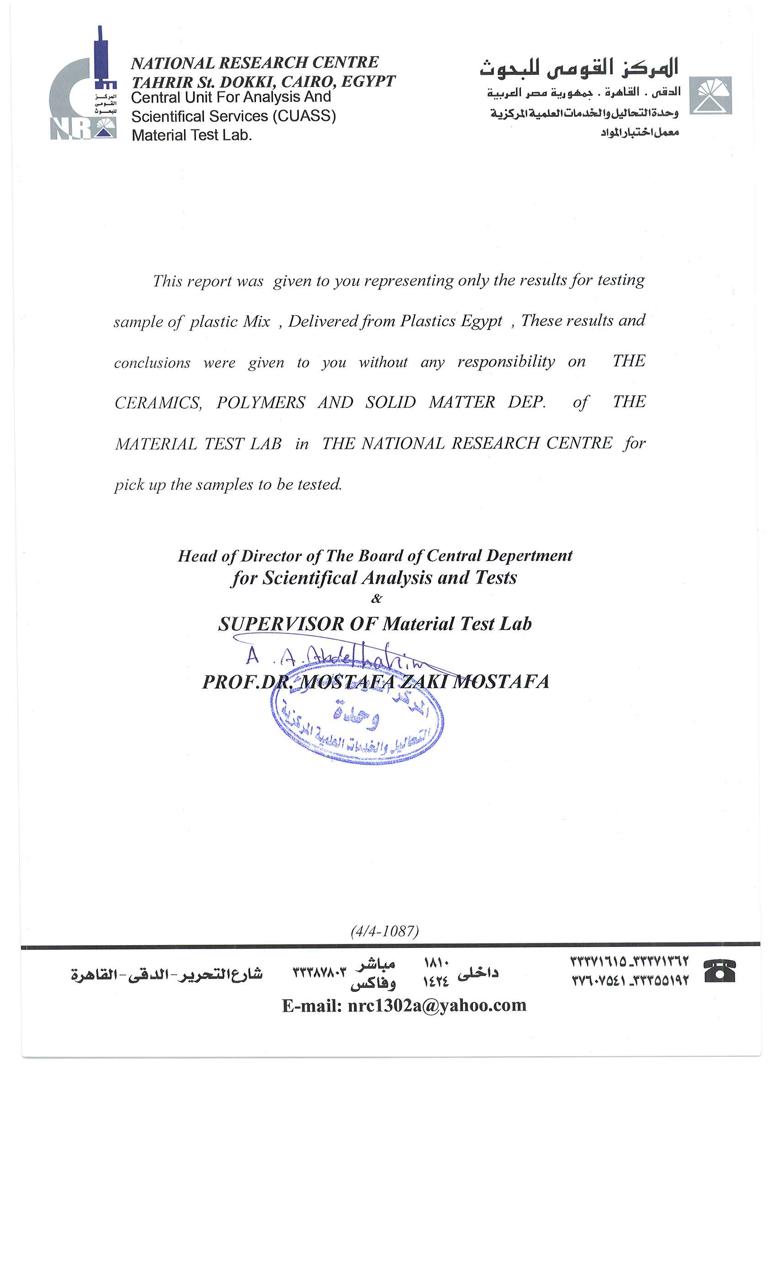


**Fig.** *A1:* A certificate stating that the tests were conducted at the National Research Center.

| 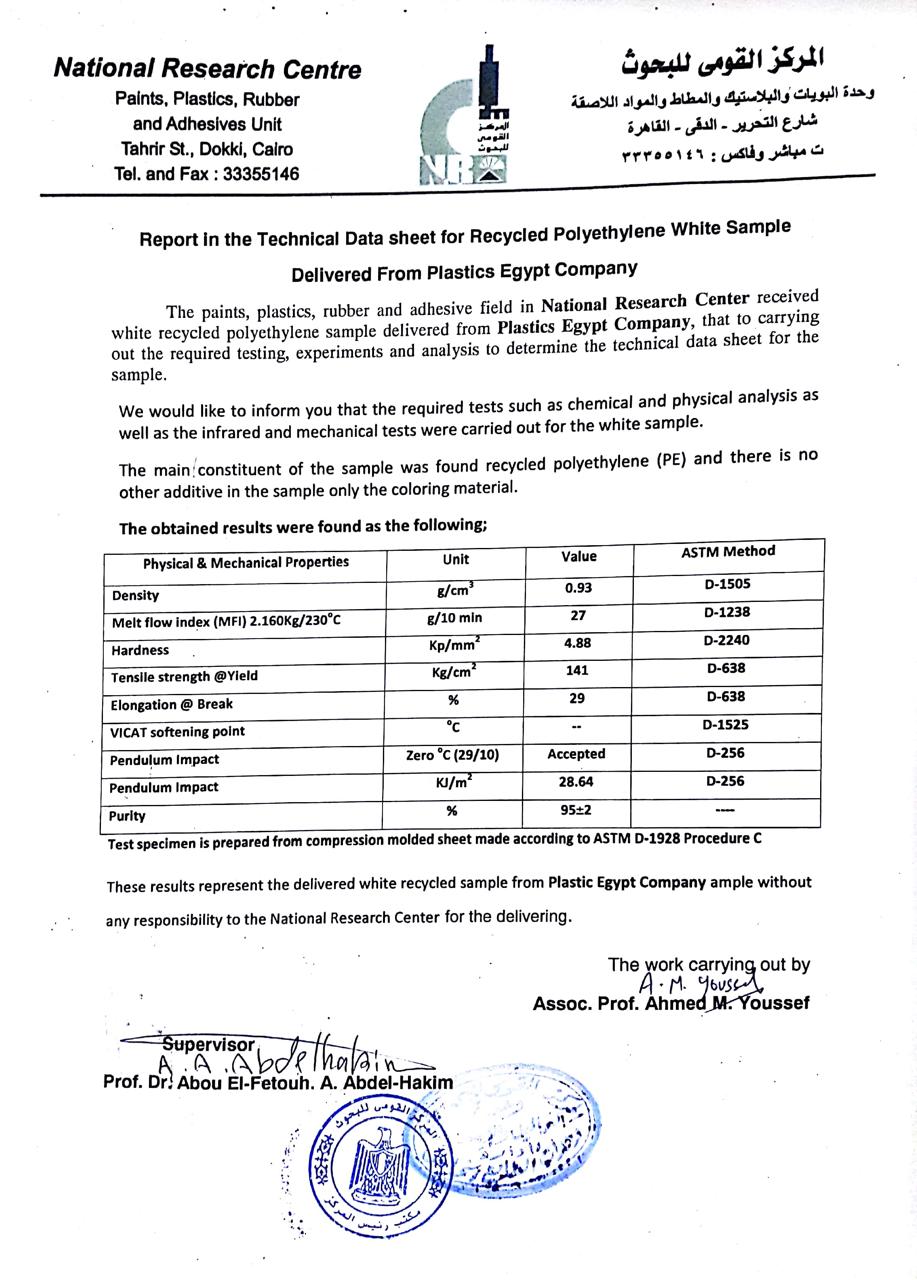 |
| --- |
| *Fig. A2: Laboratory analysis and tests carried out on plastic brick samples at the National Research Centre. Source: Authors.* |


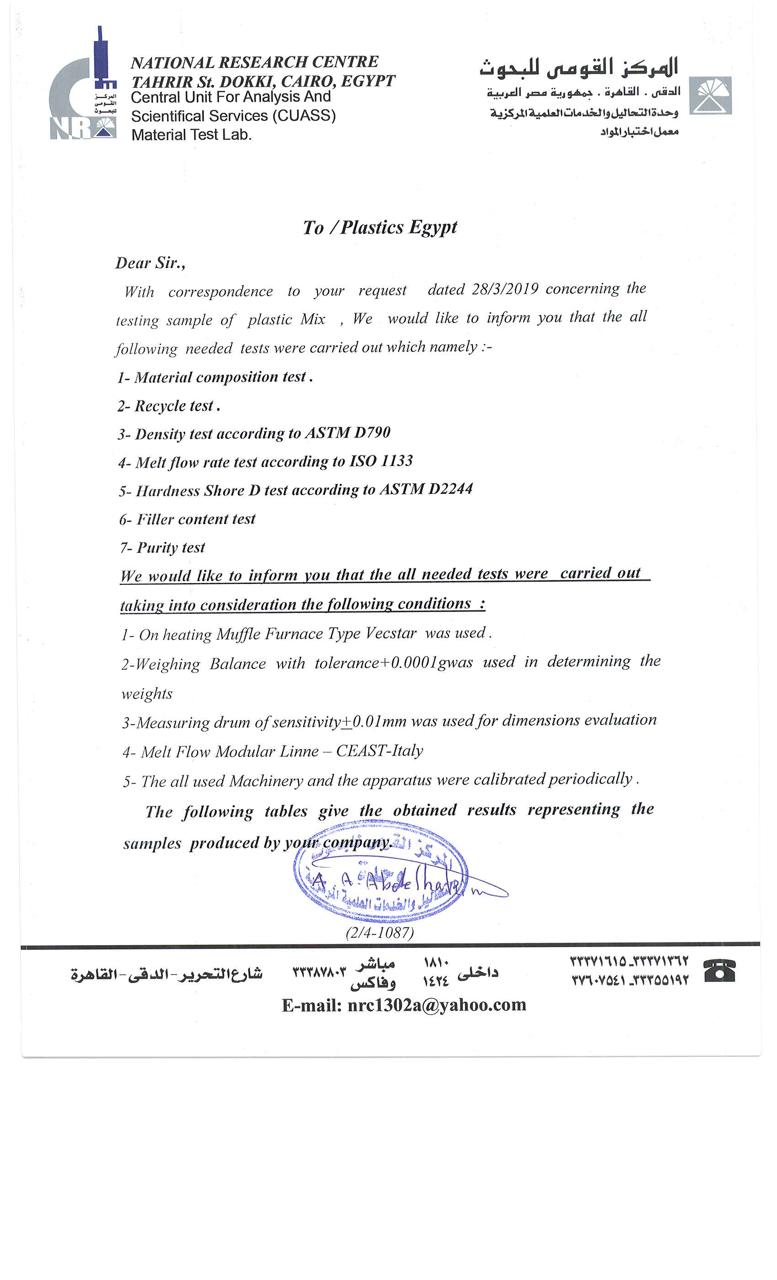


**Fig.** *A3*: Analysis of plastic brick samples and conducting tests on them at the National Research Center
